# Our Battle and Our Foes

**Published:** 1875-05

**Authors:** 


### OUR BATTLE AND OUR FOES.

WE who are striving for a better life for the masses, who are laboring from year to year to convince the people that misery must follow the violations of law ; that no debts are more surely put in course of collection than those which are charged up against us in the form of over drafts upon our stock of vitality ; that the stomach cannot be insulted and abused, without complaint ; that rational living is the only true life, *we* know what it is to battle with selfishness and indolence, and uncleanness, and sluggishness, and all the gloomy powers of ignorance, and doubt and indifference. These serried hosts are in possession ; they have held the human fortress all along through the ages of groping and darkness ; they fight decency, and vigor, and cleanliness, under the prestige of thousands of years of barbaric possession.

How we do have to talk, and work, and fight to get a lodgment, in some natures, for a sensible thought ! How many times and in how many ways do we have to utter a simple truth which runs counter to established prejudice or dense ignorance ?

How wise, how wonderfully wise, men are ! How deep, how all-embracing is their knowledge ! Not one in ten thousand can be taught anything. Tell the man who drinks ale with his dinner that the process of digestion is surely interfered with by that act, and he will swell up and turn red in the face with indignation. The alcohol in the ale stimulates his brain and warms his stomach, and he really imagines that it has done him good—poor soul. *Absolutely, positively,* in every instance, it has done him harm ; but he will laugh at you if you tell him so. Tell the unclean person who burns the most nauseous of all weeds in his mouth that he is poisoning himself, and he will pity you for an idiot. He knows better—simply because he is ignorant and prejudiced. He will tell you that smoking assists digestion ; when the truth is, the powerful narcotic simply palzies the digestive functions to such an extent that they lack sensitiveness to the wrongs imposed upon them.

Still we fight on, and still we induce desertions from the enemy's polluted camp. Day after day we are rejoiced at the sight of a little band of welcome recruits. Some days they number more than a hundred ; on other days barely half a score. Their abandonment of error and their adoption of a pure life come to us, substantially, in these words : "Send me your JOURNAL." Often they will tell us of the good which the perusal of a fugitive number, bought at some news stand, has done them. Not unfrequently they are full of thanks for some word in season which we have spoken, and which has chanced to apply to their particular case.

So we gather encouragement from day to day. We know the power of our antagonists. We know that Ignorance and Prejudice are twin giants that renew their life upon each other. We know that they are no new-fledged foes, but are as old as chaos, and that their thick armor of stolidity and conceit can only be penetrated by repeated blows. And so we hammer away, and compel many an old folly and many an old wickedness to bite the dust. What new friends will come to our aid in this work of illuminating the dark places and making deserts blossom as the rose ?

---

The fool says, I can leave off drinking whenever I please.

---

If you want a sour stomach, drink wine.

